# Prognostic value of lncRNA CBR3-AS1 for patients with cancer: A meta-analysis

**DOI:** 10.1097/MD.0000000000040361

**Published:** 2024-11-15

**Authors:** Jun Peng, Daidong Wang, Shixue Liu

**Affiliations:** a Department of Spine Surgery, Shenzhen Hospital of Integrated Traditional Chinese and Western Medicine, Shenzhen, P.R. China.

**Keywords:** cancer, lncRNA CBR3-AS1, meta-analysis, prognosis

## Abstract

**Background:**

Several studies have shown that the long noncoding RNA (lncRNA) CBR3-AS1 is overexpressed in various cancers and is playing an oncogene role. This meta-analysis aims to elucidate the relationship between lncRNA CBR3-AS1 expression and the prognosis and clinicopathological features of cancer patients.

**Methods:**

A comprehensive and systematic search was conducted in PubMed, Web of Science, Cochrane Library, and EMBASE database. Pooled odds ratios (ORs) and hazard ratios (HRs) with 95% confidence intervals (CIs) were employed to evaluate the association between lncRNA CBR3-AS1 expression and clinical outcomes and clinicopathological features in cancer patients.

**Results:**

This meta-analysis finally enrolled 9 studies comprising 800 cancer patients. The combined results indicated that lncRNA CBR3-AS1 overexpression was significantly associated with shorter overall survival (pooled hazard ratios = 1.69, 95% CI 1.28–2.21, *P* < .001). Furthermore, elevated lncRNA CBR3-AS1 expression was closely correlated with larger tumor size (large vs small OR = 2.17, 95% CI: 1.50–3.14, *P* < .0001), lymph node metastasis (yes vs no OR = 2.75, 95% CI: 1.67–4.51, *P* < .0001), distant metastasis (yes vs no OR = 3.08, 95% CI: 1.82–5.23, *P* < .0001), and advanced tumour, node, metastasis stage (III/IV vs I/II OR = 2.82, 95% CI: 1.68–4.75, *P* < .0001).

**Conclusion:**

Upregulated expression of lncRNA CBR3-AS1 showed significant association with unfavorable survival and indicated worse clinicopathological outcomes in multiple kinds of human cancer, and therefore might serve as a promising prognosis biomarker and therapeutic target for cancers.

## 1. Introduction

Cancer remains the leading cause of death worldwide, posing a major public health challenge.^[1,2]^ Even though significant advances have been made in diagnosis and therapeutic techniques in recent years, the prognosis for many patients with advanced cancer remains poor.^[3]^ The lack of effective biomarkers as therapeutic targets and prognostic indicators is considered to be a primary reason for this predicament.^[4]^ Consequently, researchers have focused on identifying optimal biomarkers for human cancers.^[5]^

Long noncoding RNAs (lncRNAs), defined as RNA molecules longer than 200 nucleotides, is an important member of the noncoding RNA family.^[6]^ Although most lncRNAs cannot encode proteins, they are actively involved in most cellular and physiological processes by acting as posttranscriptional regulators, enhancers, splicing regulators, and chromosomal modifiers.^[7]^ Numerous studies have demonstrated that lncRNAs play an important role in the pathogenesis and prognosis of malignant tumors, as well as in diverse biological processes such as tumor cell proliferation, invasion, and metastasis.^[8]^ Consequently, lncRNAs hold promise as biomarkers and therapeutic targets for cancer.^[9]^

LncRNA CBR3-AS1, also known as PlncRNA-1 or prostate cancer regulatory long noncoding RNA 1), is located in the antisense region of carbonyl reductase 3 (CBR3). It was initially identified as being overexpressed in prostate cancer tissues and cell lines compared to corresponding normal controls.^[10]^ In recent years, elevated expression of lncRNA CBR3-AS1 has been observed in various cancer types, and its overexpression has been associated with prognostic significance.^[11–19]^

It has been found that abnormal expression of lncRNA is cancer-specific and can be detected in cancer samples, blood, tissue fluid, and bodily fluids, thus it has great potential to become a tumor marker and can be used for noninvasive early cancer screening and diagnosis in clinical applications. There is currently no relevant study that has systematically reviewed the relationship between lncRNA CBR3-AS1 expression and prognosis. Therefore, this study aims to investigate the relationship between lncRNA CBR3-AS1 expression and prognosis in cancer patients.

## 2. Methods

### 2.1. Publication search

Web of Science, PubMed, Embase, Cochrane Library were used for the literature search, up until April 15th, 2024. The search strategy which was used in PubMed is as follows: (((((“Neoplasms”[Mesh]) OR (((((((((((((((((Tumor[Title/Abstract]) OR (Neoplasm[Title/Abstract])) OR (Tumors[Title/Abstract])) OR (Neoplasia[Title/Abstract])) OR (Neoplasias[Title/Abstract])) OR (Cancer[Title/Abstract])) OR (Cancers[Title/Abstract])) OR (Malignant Neoplasm[Title/Abstract])) OR (Malignancy[Title/Abstract])) OR (Malignancies[Title/Abstract])) OR (Malignant Neoplasms[Title/Abstract])) OR (Neoplasm, Malignant[Title/Abstract])) OR (Neoplasms, Malignant[Title/Abstract])) OR (Benign Neoplasms[Title/Abstract])) OR (Benign Neoplasm[Title/Abstract])) OR (Neoplasms, Benign[Title/Abstract])) OR (Neoplasm, Benign[Title/Abstract]))))) AND ((lncRNA CBR3-AS1) OR (PlncRNA-1)).

### 2.2. Inclusion and exclusion criteria

The inclusion criteria were established as follows: (1) study subjects must be cancer patients; (2) the study must report the association between lncRNA CBR3-AS1 expression and available survival data as well as clinical histological features; (3) cancer patients should be categorized into low and high expression groups based on lncRNA CBR3-AS1 expression levels. The exclusion criteria included: (1) reviews, letters, conference abstracts, and animal studies; (2) studies lacking overall survival data or clinicopathological parameters; (3) duplicate publications.

### 2.3. Data extraction and quality assessment

Daidong Wang and Shixue Liu independently reviewed each eligible study and extracted the relevant data. The data extraction from the included articles encompassed the following details: the last name of the first author, publication year, cancer type, country of origin, sample size, source of samples, number of patients, detection methods, study endpoints, follow-up duration, outcome measures, and clinicopathological parameters. In cases where both univariate and multivariate analyses were conducted using the Cox regression model, multivariate analysis was prioritized due to its greater accuracy in addressing confounding factors. If hazard ratios (HRs) and 95% confidence intervals (CIs) or overall survival (OS) data were unavailable in the articles, information was indirectly obtained from Kaplan–Meier survival curves.^[20]^ The quality of the included articles was assessed using the Newcastle–Ottawa Scale, with articles scoring ≥ 6 classified as high quality.

### 2.4. Statistical analysis

STATA software version 11.0 (Stata, College Station, TX) and Review Manager version 5.4 (The Cochrane Collaboration, Copenhagen, Denmark) were utilized for data analysis of the included studies. HRs and 95% CIs were pooled to assess the association between lncRNA CBR3-AS1 expression and survival outcomes. Additionally, pooled odds ratios (ORs) and 95% CIs were used to evaluate the relationship between lncRNA CBR3-AS1 expression and clinical parameters. Heterogeneity in the meta-analysis was assessed using the *I*² and *Q* tests. If *I*² < 50% or *P* > .05, indicating no significant heterogeneity, a fixed-effects model was applied; otherwise, a random-effects model was selected.^[21,22]^ Publication bias was examined through Egger and Begg funnel plots, and sensitivity analysis was conducted to evaluate the stability of the results.^[23]^ *P* < .05 was recognized as statistical significance.

## 3. Results

### 3.1. Study selection and characteristics

**Figure 1.**
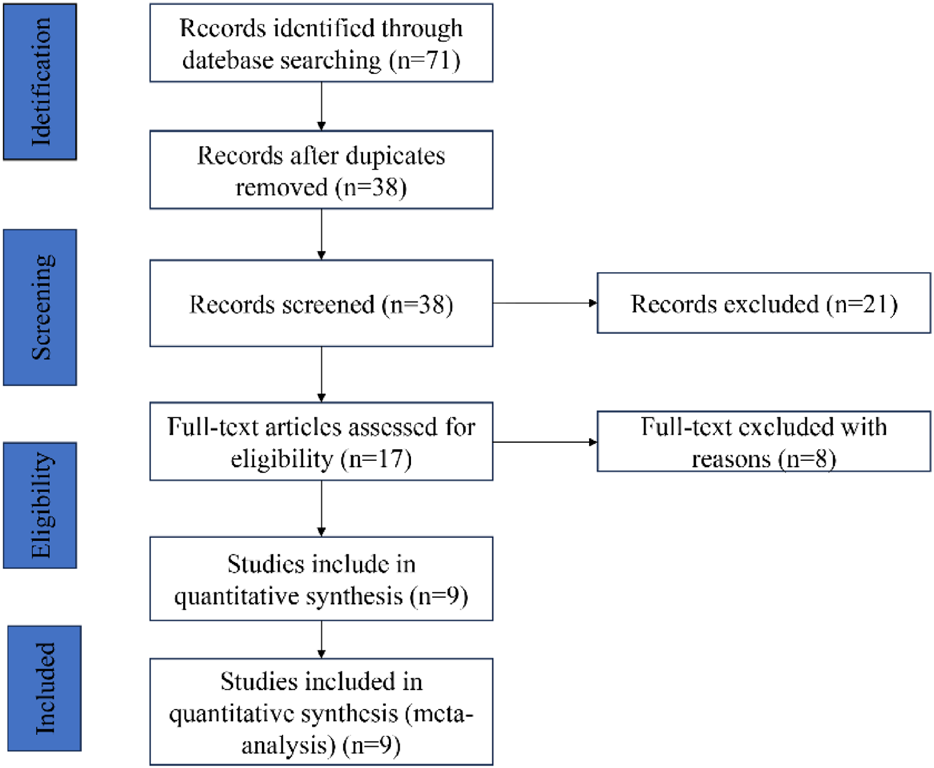
Literature screening and study selection process flow diagram.

The literature selection process is illustrated in Figure 1. Initially, 71 articles were retrieved from the 4 mentioned databases. After removing duplicates and excluding studies with irrelevant topics, further screening of titles, abstracts, and full texts was conducted. Ultimately, 9 studies were included in the meta-analysis. All of the selected studies were conducted in China, with a combined total of 800 patients and sample sizes ranging from 56 to 133. These studies were published between 2018 and 2022 and involved 7 different cancer types, including cervical cancer,^[19]^ colorectal cancer,^[11,12]^ breast cancer,^[13,14]^ glioma,^[15]^ non‑small‑cell lung cancer,^[16]^ lung adenocarcinoma,^[17]^ and osteosarcoma.^[18]^ All of the included studies employed real-time quantitative PCR to measure lncRNA CBR3-AS1 expression, with patients classified into high and low expression groups based on the expression levels of lncRNA CBR3-AS1. None of the articles had a Newcastle–Ottawa Scale score below 6, indicating that all included studies are of high quality. This meta-analysis has been registered on the PROSPERO database under the registration number CRD42023485769. The detailed characteristics of all eligible studies are presented in Table 1.

**Table 1:** Characteristics of enrolled studies in this meta-analysis.

First author	Year	Country	Cancer type	Expression (high/low)	Sample size	Sample source	Detection methods	Study endpoints	Follow time (months)	HR availability	NOS score
Cai	2022	China	CC	28/28	56	Tissue	RT‐qPCR	OS	60	K–M curve	8
Guan	2020	China	NSCLC	29/28	57	Tissue	RT‐qPCR	OS	60	K–M curve	8
Hou	2021	China	LUAD	37/38	75	Tissue	RT‐qPCR	OS	100	K–M curve	9
Song	2018	China	CRC	39/38	77	Tissue	RT‐qPCR	OS	60	K–M curve	9
Wang	2018	China	Glioma	52/52	104	Tissue	RT‐qPCR	OS	36	K–M curve	9
Xie	2022	China	CRC	63/70	133	Tissue	RT‑qPCR	OS	60	K–M curve	9
Xu	2019	China	BC	37/33	70	Tissue	RT‐qPCR	OS	72	K–M curve	9
Zhang	2018	China	OS	66/66	132	Tissue	RT‐qPCR	OS	72	Multivariate analysis	9
Zhang	2021	China	BC	54/42	96	Tissue	RT-qPCR	OS	120	K–M curve	9

*Note*: BC = breast cancer, CC = cervical cancer, CRC = colorectal cancer, K–M curve = Kaplan–Merier survival curve, LUAD = lung adenocarcinoma, NOS = Newcastle–Ottawa scale, NR = not reported, NSCLC = non‑small‑cell lung cancer, OS = osteosarcoma, RT‑qPCR = real-time quantitative PCR (RT-qPCR).

### 3.2. Association between lncRNA CBR3-AS1 expression and prognosis

**Figure 2.**
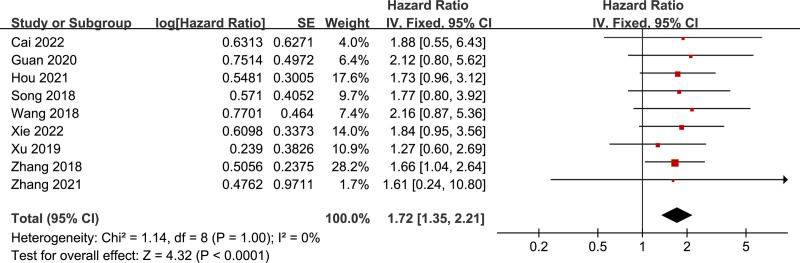
Forest plot for the relation between lncRNA CBR3-AS1 expression and overall survival based on different types of cancers.

The HR from 9 eligible studies, comprising 800 patients, was pooled to examine the relationship between lncRNA CBR3-AS1 expression and OS. No statistically significant heterogeneity was detected among these studies (*I*² = 0%, *P* = 1.00), allowing for the use of fixed-effects models. The combined HR was 1.72 (95% CI: 1.35–2.21, *P* < .0001) (Fig. 2), indicating that patients with high lncRNA CBR3-AS1 expression had significantly worse OS compared to those with low expression. These findings suggest that elevated lncRNA CBR3-AS1 expression holds promise as a predictive marker for poorer overall survival in cancer patients.

### 3.3. Associations between lncRNA CBR3-AS1 expression and clinicopathological parameters

**Figure 3.**
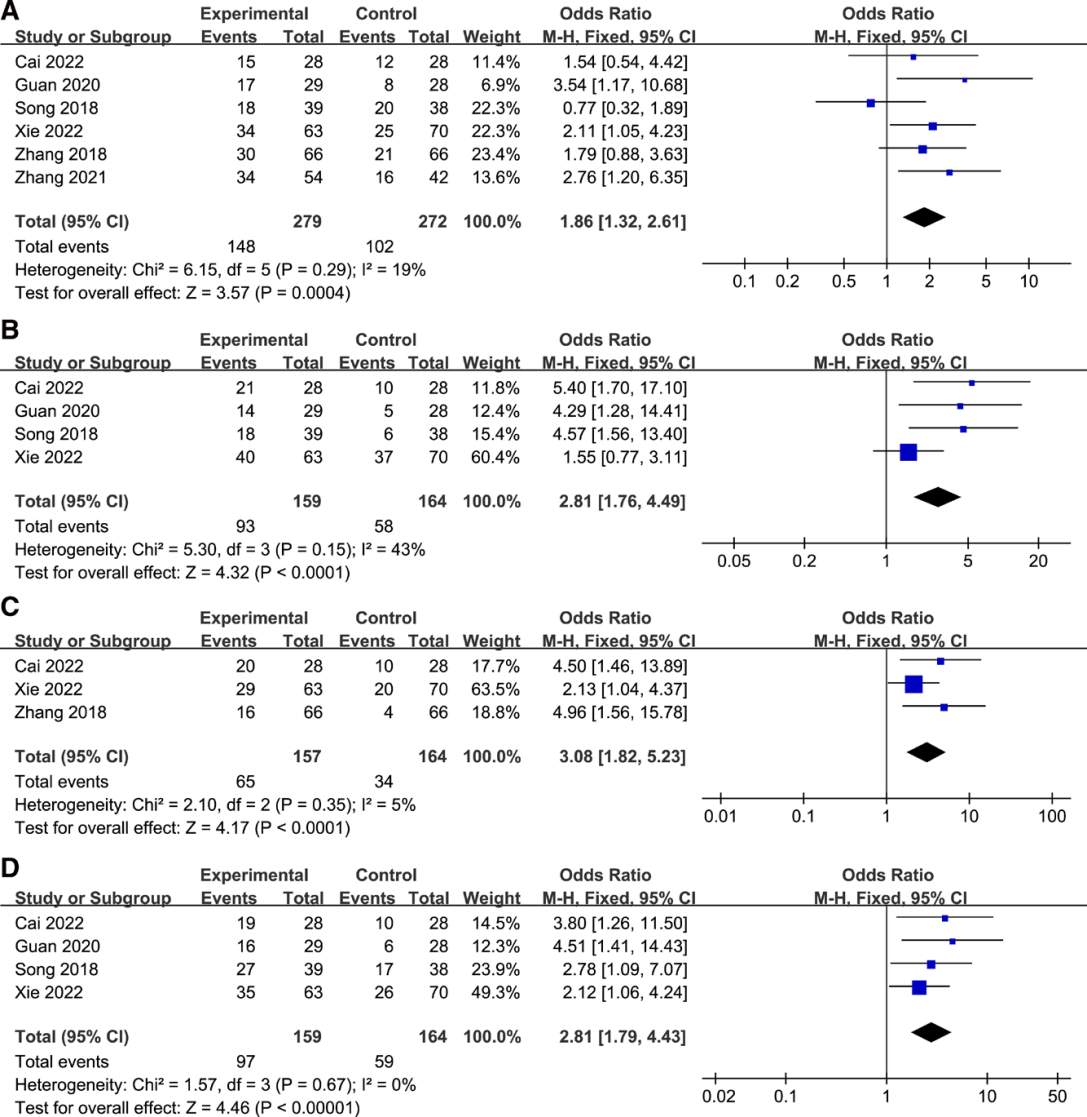
Forest plot for the relation between lncRNA CBR3-AS1 expression and clinicopathological characteristics: (A) Tumor size; (B) LM; (C) DM; (D) TNM. DM = distant metastasis, LM = lymph node metastasis, TNM = tumour, node, metastasis.

The OR and 95% CI were used to assess the relationship between lncRNA CBR3-AS1 expression and clinicopathological features as shown in Figure 3 and Table 2. Patients with high lncRNA CBR3-AS1 expression tended to have larger tumor size (large vs small OR = 1.86, 95% CI: 1.32–2.61, *P* = .0004), more susceptible to lymph node metastasis (yes vs no OR = 2.81, 95% CI: 1.76–4.49, *P* < .0001), distant metastasis (yes vs no OR = 3.08, 95% CI: 1.82–5.23, *P* < .0001), and higher tumour, node, metastasis (TNM) stage (III/IV vs I/II OR = 2.81, 95% CI: 1.79–4.43, *P* < .00001).

**Table 2:** Meta-analysis results for the association between lncRNA CBR3-AS1 expression and clinicopathological characteristics.

Variables	Studies (n)	Patient (n)	Pooled OR (95% CI)	*P*_Het_	*I*² (%)	*P*	Model
Age (old: young)	6	551	1.08 [0.77, 1.51]	.41	2	.67	Fixed
Gender (male: female)	4	399	1.26 [0.84, 1.89]	.99	0	.25	Fixed
Tumor size (large: small)	6	551	1.86 [1.32, 2.61]	.29	19	.0004	Fixed
Differentiation (poor: well)	3	267	1.71 [1.00, 2.92]	.78	0	.05	Fixed
Lymph node metastasis (yes: no)	4	323	2.81 [1.76, 4.49]	.15	43	<.0001	Fixed
Distant metastasis (yes: no)	3	321	3.08 [1.82, 5.23]	.35	5	<.0001	Fixed
TNM stage (III/IV: I/II)	4	323	2.81 [1.79, 4.43]	.67	0	<.00001	Fixed

Fixed = fixed-effects model, Het = heterogeneity, OR = odds ratio, TNM = tumour, node, metastasis.

However, there was no significant correlation between lncRNA CBR3-AS1 expression and tumor differentiation (poor vs well OR = 1.71, 95% CI:1.00–2.92, *P* = .05), age (old vs young OR = 1.08, 95% CI:0.77–1.51, *P* = .67) and gender (male vs female OR = 1.26, 95% CI:0.84–1.89, *P* = .25) (Figure S1, Supplemental Digital Content, http://links.lww.com/MD/N895). In addition, Funnel plots indicate publication bias did not affect the pooled results in the meta-analysis (Figure S2, Supplemental Digital Content, http://links.lww.com/MD/N896).

### 3.4. Publication bias and sensitivity analysis

**Figure 4.**
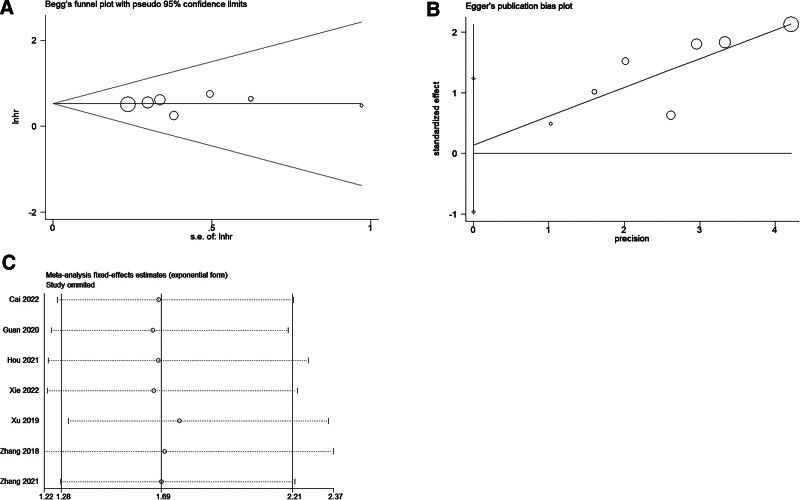
Begg funnel plot, Egger funnel plot and Sensitivity analysis for the evaluation of potential publication bias in the impact of lncRNA CBR3-AS1 on overall survival: (A) Begg funnel plot; (B) Egger funnel plot; (C) Sensitivity analysis.

The Begg and Egger tests were employed to assess potential publication bias in the analysis of lncRNA CBR3-AS1 and OS. The results indicated no significant publication bias (Begg test: *P* = .466; Egger test: *P* = .519), a conclusion further supported by the funnel plots from both tests (Fig. 4A and B). Moreover, sensitivity analysis demonstrated that the HR remained stable when any of the included studies were excluded, suggesting the robustness of the findings (Fig. 4C).

## 4. Discussion

Cancer is one of the leading causes of death worldwide and, despite significant advances in diagnostic and therapeutic technologies, numerous challenges persist. Traditional diagnostic methods suffer from insufficient sensitivity and specificity in some cases, while therapeutic tools such as surgery, chemotherapy, radiotherapy, and immunotherapy, despite improving survival rates, are accompanied by treatment resistance and side effects.^[24,25]^ Therefore, the search for new diagnostic and prognostic markers is an important direction in current cancer research. The roles of lncRNAs in cancer are extensive and complex, and they are involved in tumourigenesis, progression, and metastasis through the regulation of gene expression, cell proliferation and apoptosis, cell migration and invasion, epigenetic modifications, signaling pathways, and drug resistance. Studying the functions and mechanisms of lncRNAs not only contributes to an in-depth understanding of tumor biology but may also provide new targets and strategies for tumor diagnosis and treatment.^[6,8]^

The lncRNA CBR3-AS1 has been identified as an important lncRNA in a variety of cancers. Studies have shown that CBR3-AS1 may play an important role in tumor growth, metastasis, and patient survival. Song et al identified that the knock-down of lncRNA CBR3-AS1 significantly suppressed colorectal cancer cell proliferation, migration, and invasion, while high lncRNA BLACAT1 expression served as a poor prognostic factor for colorectal cancer cell patients.^[11]^ Zhang et al found that lncRNA CBR3-AS1 was highly expressed in osteosarcoma tissues and cell lines and correlated with Enneking stage, distant metastasis, and histological grading. Survival analysis showed that high expression of lncRNA CBR3-AS1 was an independent poor prognostic factor for osteosarcoma patients. Loss-of-function studies showed that knock-down of lncRNA CBR3-AS1 inhibited proliferation, migration, invasion of osteosarcoma cells and promoted apoptosis, but had no effect on cell cycle distribution.^[18]^ Zhang et al showed that lncRNA CBR3-AS1 may be an important biomarker for evaluating the prognosis of breast cancer patients, and lncRNA CBR3-AS1 can increase the resistance of breast cancer to ADR by competitively binding miR-25-3p with JNK1/MEK4 and enhancing the MAPK signaling pathway.^[14]^ In summary, lncRNA CBR3-AS1 plays an important role in tumors and deserves further analysis and statistics to clarify its specific function in tumors.

To further clarify the role of lncRNA CBR3-AS1 across various cancers, a meta-analysis was conducted to assess the impact of its aberrant expression on the prognostic significance and clinicopathological characteristics of cancer patients. This meta-analysis included 9 studies covering 7 different cancer types, with a combined sample of 800 patients. The findings revealed that elevated expression of lncRNA CBR3-AS1 is linked to poor survival outcomes across multiple tumor types. Additionally, overexpression of lncRNA CBR3-AS1 was associated with higher TNM stages, larger tumor sizes, lymph node metastasis, and distant metastasis. However, no significant correlation was observed between lncRNA CBR3-AS1 expression and factors such as age, gender, or tumor differentiation. Overall, high lncRNA CBR3-AS1 expression demonstrates significant prognostic value for cancer patients and may offer valuable insights for clinical diagnosis and treatment strategies.

However, several limitations exist in the current meta-analysis. First, in some of the included studies, the HR and corresponding 95% confidence interval (CI) were indirectly extracted, which could introduce potential errors. Second, all cancer patients involved were of Chinese origin, limiting the global generalizability of the findings. Third, insufficient sample sizes for specific cancer types prevented further in-depth analysis, indicating the need for additional studies to validate the conclusions drawn from this research.

## 5. Conclusions

In conclusion, this study demonstrates that elevated expression of lncRNA CBR3-AS1 may be linked to reduced overall survival in cancer patients, as well as associations with lymph node metastasis, advanced TNM stage, and increased tumor size. Consequently, lncRNA CBR3-AS1 holds potential as a prognostic biomarker for various cancers. Future investigations, utilizing larger sample sizes, more diverse populations, and standardized clinical trials, are necessary to further evaluate the prognostic significance of lncRNA CBR3-AS1 across different tumor types.

## Acknowledgments

We are grateful to all researchers of enrolled studies.

## Author contributions

**Conceptualization:** Jun Peng, Shixue Liu.

**Data curation:** Jun Peng, Daidong Wang.

**Formal analysis:** Jun Peng, Shixue Liu.

**Funding acquisition:** Jun Peng.

**Investigation:** Jun Peng, Shixue Liu.

**Methodology:** Jun Peng, Daidong Wang.

**Project administration:** Jun Peng.

**Resources:** Jun Peng, Daidong Wang.

**Software:** Jun Peng, Daidong Wang, Shixue Liu.

**Supervision:** Jun Peng, Daidong Wang, Shixue Liu.

**Validation:** Jun Peng, Shixue Liu.

**Visualization:** Jun Peng, Shixue Liu.

**Writing – original draft:** Jun Peng, Shixue Liu.

**Writing – review & editing:** Jun Peng, Daidong Wang, Shixue Liu.

## Supplementary Material
