# Supplementary material for: Prognostic value of lncRNA CBR3-AS1 for patients with cancer: A meta-analysis
Source: Medicine (Baltimore). 2024 Nov 15;103(46):e40361. doi: 10.1097/MD.0000000000040361 (PMC11576033; doi:10.1097/MD.0000000000040361)
Supplement: Supplementary file 1 [file medi-103-e40361-s001.docx]

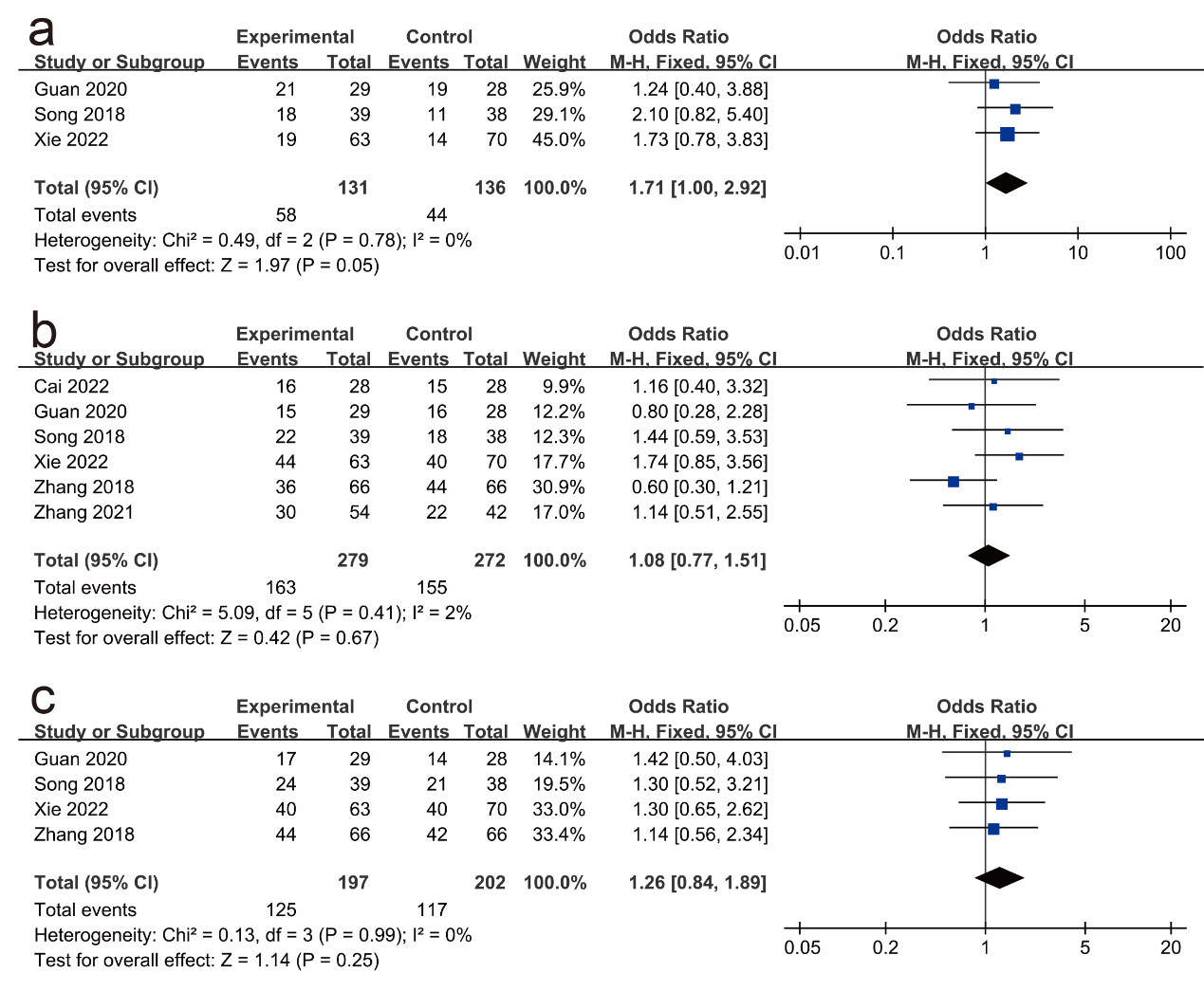


**Supplemental Digital Content (Figure S1)：Figure S1. Forest plot for the relation between lncRNA CBR3-AS1 expression and clinicopathological characteristics. a, tumor differentiation; b, age; c, gender.**
