# Supplementary material for: Prognostic value of lncRNA CBR3-AS1 for patients with cancer: A meta-analysis
Source: Medicine (Baltimore). 2024 Nov 15;103(46):e40361. doi: 10.1097/MD.0000000000040361 (PMC11576033; doi:10.1097/MD.0000000000040361)

**Supplemental Digital Content (Figure S2): Figure S2. Funnel plots for the meta-analyses of the association between lncRNA CBR3-AS1 expression and clinicopathological parameters. a, HR; b, tumor size; c, lymph node metastasis; d, distant metastasis; e, TNM stage; f, differentiation; g, age; h, gender.**


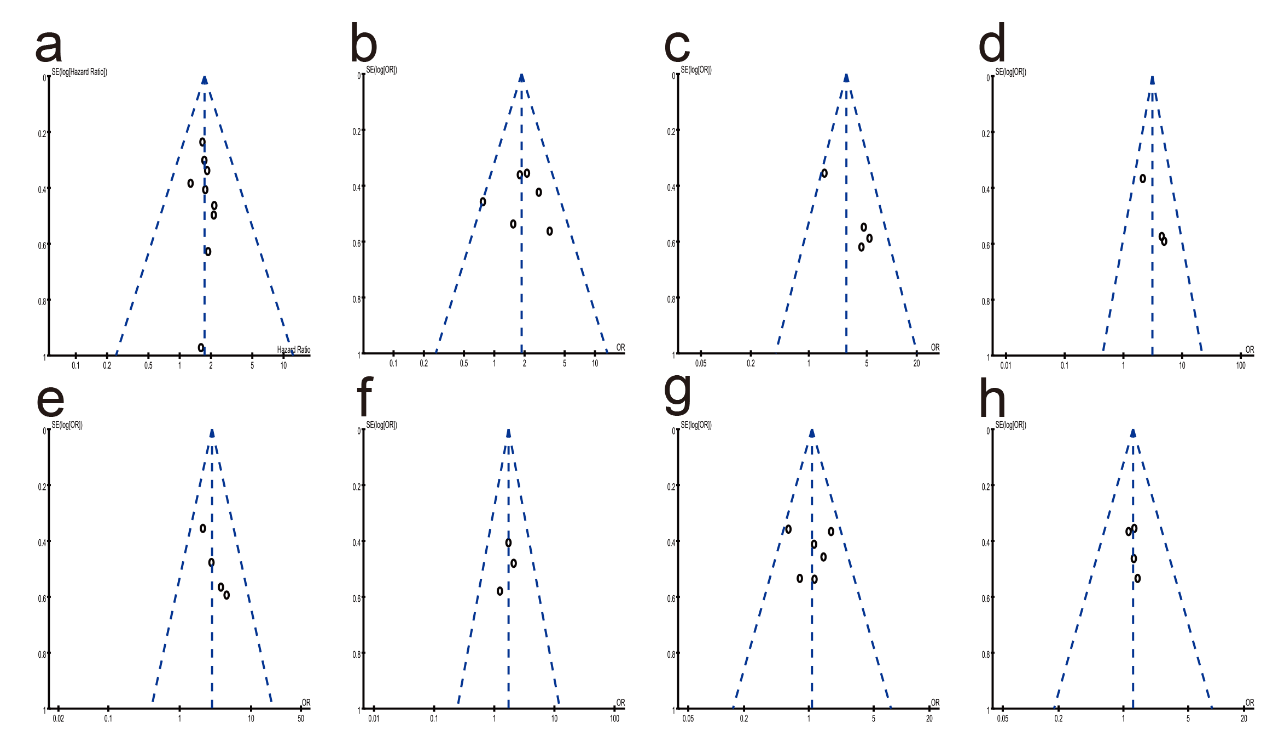

Supplement: Supplementary file 2 [file medi-103-e40361-s002.docx]
